# Medical education research in GCC countries

**DOI:** 10.1186/s12909-015-0293-6

**Published:** 2015-02-01

**Authors:** Sultan Ayoub Meo, Asim Hassan, Mansoor Aqil, Adnan Mahmood Usmani

**Affiliations:** 1 Department of Physiology, College of Medicine, King Saud University, P.O. Box 2925, Riyadh, 11461 Saudi Arabia; 2 University Diabetes Centre, King Saud University, Riyadh, Saudi Arabia; 3 Department of Anesthesia, College of Medicine, King Saud University, Riyadh, Saudi Arabia

**Keywords:** GCC, Medical Education, Research papers, Indexed Journal

## Abstract

**Background:**

Medical education is an essential domain to produce physicians with high standards of medical knowledge, skills and professionalism in medical practice. This study aimed to investigate the research progress and prospects of GCC countries in medical education during the period 1996–2013.

**Methods:**

In this study, the research papers published in various global scientific journals during the period 1996–2013 were accessed. We recorded the total number of research documents having an affiliation with GCC Countries including Saudi Arabia, Bahrain, Kuwait, Qatar, United Arab Emirates and Oman. The main source for information was Institute of Scientific Information (ISI) Web of Science, Thomson Reuters.

**Results:**

In ISI-Web of Science, Saudi Arabia contributed 40797 research papers, Kuwait 1666, United Arab Emirates 3045, Qatar 4265, Bahrain 1666 and Oman 4848 research papers. However, in Medical Education only Saudi Arabia contributed 323 (0.79%) research papers, Kuwait 52 (0.03%), United Arab Emirates 41(0.01%), Qatar 37(0.008%), Bahrain 28 (0.06%) and Oman 22 (0.45%) research papers in in ISI indexed journals. In medical education the Hirsch index (h-index) of Saudi Arabia is 14, United Arab Emirates 14, Kuwait 11, Qatar 8, Bahrain 8 and Oman 5.

**Conclusion:**

GCC countries produced very little research in medical education during the period 1996–2013. They must improve their research outcomes in medical education to produce better physicians to enhance the standards in medical practice in the region.

## Background

The Gulf Cooperation Council (GCC) countries are the Arab states of the Gulf including Saudi Arabia, Bahrain, Kuwait, Qatar, United Arab Emirates and Oman. GCC was founded in May 1981. The total area of GCC countries is 2,673,108 km^2^, with 2.1 trillion economy [1]. These countries are highly blessed with natural resources including oil and gas with high income advantages. These countries are speedily moving ahead to promote a high-quality education for the citizens and devoted special attention to fostering higher education and research in the country [2].

The excellence of healthcare depends on the competencies of the health care professionals. Medical education is the foundation for a high quality health care. It is generally agreed that where training is important there education is vital. It is this education which will enable the medical students to lead and meet the ever-changing requirements of the society. Medical practice will definitely improve by research in medical education. Research in science and medical education play a significant role in the country’s economic growth along with long-term sustainable development and eventually contribute to the improvement of living standards and quality of life [3]. Investment in research is more vital for the progress and prosperity. To recognize and quantify the research progress of a country in any subject, bibliometric indicators are essential tools to understand the growth and global spread of research. These indicators are mainly based on the number of scientific research documents published and their visibility in global science [4]. Scientific publications are a key indicator of the development of a country, and measuring the research output provides information that forms the basis of strategic decisions [5]. The research pursuing behavior is essential in economic success of a country [6]. In order to achieve long-term and sustainable economic growth, spending on research and development is essential to produce a substantial amount of innovative research. There is a direct relationship between research and the development of individual states. GCC countries are fostering the research to promote education and research culture in the region.

The history of medical education starts with the Abraham Flexner’s report on the state of medical education in the United States and Canada published in 1910. With the passage of time, the introduction of medical education was expanded at large number of medical schools, and this change was noticed without geographical boundaries. However, the available literature shows that a number of publications on medical education are available in the scientific databases starting from 1996. Hence, this study aimed to investigate the research outcome of GCC countries in medical education during the period 1996–2013.

## Methods

This observational study was conducted in the Department of Physiology, College of Medicine, King Saud University, Riyadh, Saudi Arabia. In this study, we reviewed the research publications in medical education published in ISI indexed journals. The data about number of universities were collected from the World Association of Universities [7]. The information regarding scientific journals indexed in Institute of Scientific Information (ISI) was obtained from Web of Science, Institute of Scientific Information (ISI) Journal Citation Reports, Thomson Reuters [8]. Data for research documents in medical education published in ISI journals during the period 1996–2013 were obtained from ISI web of science [8]. Data for research papers published in ISI indexed journals only were obtained from the Institute of Scientific Information (ISI) Web of Science [8]. For ISI indexed journals, we logged on to Web of Science, the territory was selected, GCC country name “Saudi Arabia” was entered, and the names of journals along with impact factors for each journal were retrieved. For the recording of bibliometric indicators, research outcome in all world scientific journals indexed in ISI web of Science, region and country was selected, subject field “Medical Education” was opted and detailed information regarding the bibliometric indicators including total number of research papers (documents), total citations, citations per document and Hirsch Index (*h*-index) [9] in medical education were obtained.

### Ethics statement

In this study we reviewed the data base literature on medical education in GCC countries hence, we did not require the ethical approval.

### Statistical analysis

The data were analyzed by using Statistical Package for the Social Sciences (SPSS) Inc Chicago, IL, USA, software version 18. Data were expressed as Mean ± Standard Error of Mean (SEM). The Pearson correlation coefficient was calculated to find the strength of relation between different variables. *p*-value <0.05 was considered significant.

## Results

In GCC countries the total number of universities and degree awarding institutes are 126 and from them 40 are medical universities. There are a total of 24 scientific journals which are indexed in a Journal Citation Report, Thomson Reuters Institute of Scientific Information (ISI). The total number of research papers published from GCC countries in ISI indexed journals during the period 1996–2013 is 56283 and GCC countries Hirsch Index (*h*-index) is 446 (Table 1).

**Table 1:** **Total number of universities, medical universities, ISI Indexed journals, publications in ISI web of science and Herish Index of GCC countries during the period 1996–2013** [8]

Country	Total Number of universities	Total number of medical universities	ISI indexed journals	Publication in ISI-Web of science	H-Index of all publications
Saudi Arabia	63	30	7	40797	124
United Arab Emirates	33	5	12	3045	87
Kuwait	6	1	3	1666	83
Qatar	12	1	0	4265	50
Bahrain	11	1	2	1666	39
Oman	11	2	0	4844	63
Total	126	40	24	56283	446

Ref: The data was recorded from ISI-web of Science [8].

Table 2 shows the total number of publication in medical education, Herish-index, total number of citations and citations per documents in medical education in ISI web of science of GCC countries during the period 1996–2013. Total number of publications contributed by the GCC countries in medical education are 503, total citation 2261; citations per documents 5.99, and the country’s Hirsch Index in medical education based documents (*h*-index) is 59. Based on the number of research publications in medical education Saudi Arabia is standing first in GCC countries (Table 2).

**Table 2:** **Total number of publication, number of citations, citations per documents in medical education in ISI web of science and Herish-index of GCC countries** [9]

Country	Publications in medical education in ISI-Web of science	Total citation of medical education publications	Citations per documents of medical education papers	H-Index based on medical education publications
Saudi Arabia	323	1025	3.17	14
United Arab Emirates	41	477	11.63	14
Kuwait	52	308	5.92	11
Qatar	37	205	5.54	8
Bahrain	28	155	5.54	8
Oman	22	91	4.14	5
Total	503	2261	35.94	59

Ref: The data was recorded from ISI-web of Science and SCI-mago report [8,9].

Table 3 shows the Pearson correlation coefficient between GDP per capita, spending on R&D as % of total GDP, number of medical schools/medical universities and total number of research papers in medical education in ISI web of Science among GCC countries. There was a positive correlation between number of medical schools and universities with total number of research papers appeared in ISI web of science (r^2^ = 0.096, *p* = 0.0001). Moreover, this correlation was also positively established between the number of medical schools and universities and total number of research documents published only in medical education subject (r^2^= = 0.986, *p* = 0.0001).

**Table 3:** **Pearson correlation coefficient between GDP per capita, spending on R&D as % of total GDP, number of medical schools/medical universities and total number of research publication and medical education publications in ISI web of Science among GCC countries**

Parameters	Total number of publication in Web of Science	Medical Education Publications in Web of Science
**GDP per capita US$**	r^2^ = 0.108	r^2^ = 0.099
	p = 0.524	p = 0.543
**Spending on R&D**	r^2^ = 0.106	r^2^ = 0.080
	p = 0.529	p = 0.585
**Medical Schools/Medical Universities**	r^2^ = 0.976	r^2^ = 0.986
	p = 0.0001	p = 0.0001

However, there was no association between GDP per capita and total number of research documents (r^2^= = 0.108, *p* = 0.524), medical education publications (r^2^= = 0.999, *p* = 0.543).

Moreover, there was no association between Spending on R&D and total number of research documents (r^2^ = 0.106, *p* = 0.529), and medical education publications (r^2^ = 0.080, *p* = 0.585) among GCC countries (Table 3).

## Discussion

**Figure 1:**
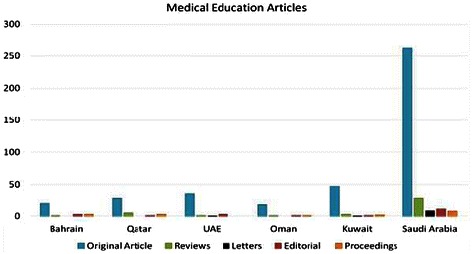
**Number of original articles, review articles, letters, editorials and proceedings published in medical education in ISI indexed journals from GCC countries.**

**Figure 2:**
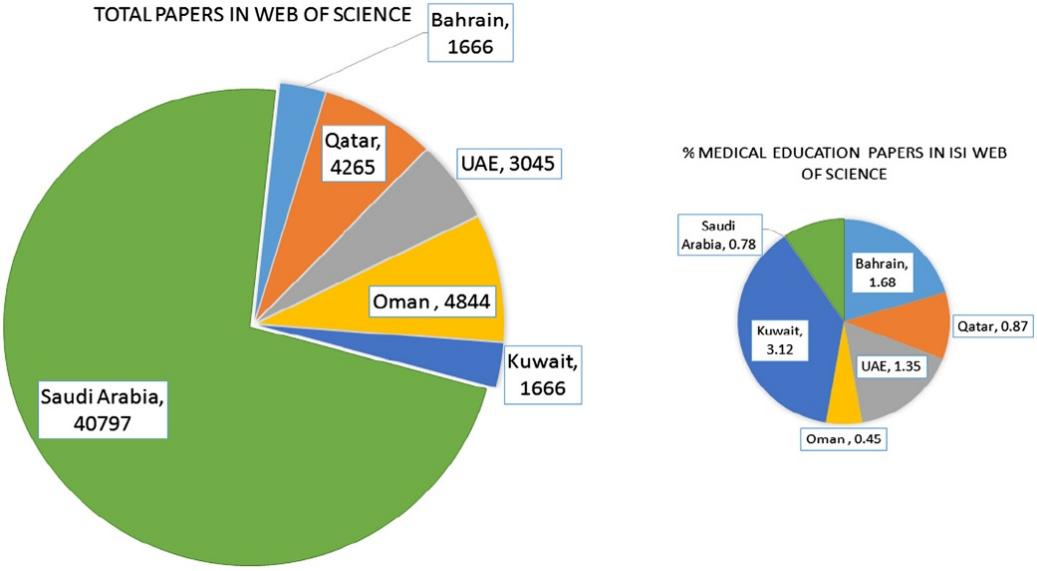
**Number of research documents published in ISI web of science and number of research papers published in medical education with their percentage in GCC countries.**

In the present study, we investigated the progress and prospects of GCC-countries’ research in medical education during the period 1996–2013. We found that, among GCC countries, Saudi Arabia was the highest contributor to global science with 40797 research papers followed by Kuwait 1666, United Arab Emirates 3045, Qatar 4265, Bahrain 1666 and Oman 4848 research papers (Figure 1). However, in Medical Education, Saudi Arabia contributed 323 (0.79%) research papers, Kuwait 52 (0.03%), United Arab Emirates 41(0.01%), Qatar 37(0.008%), Bahrain 28 (0.06%) and Oman 22 (0.45%) research papers in ISI indexed journals (Figure 2). In medical education the Hirsch index (*h*-index) of Saudi Arabia is 14, United Arab Emirates 14, Kuwait 11, Qatar 8, Bahrain 8 and Oman 5.

In GCC, the current trend in medical education reflects major modifications in educational paradigms in the context of swift shifting and increasing demands in the health care delivery system. Since the last few years, GCC countries have continuously been increasing funding for education and research. The education budget of Saudi Arabia was $32.62 billion in 2009, $36.63 billion in 2010 and $45.18 billion in 2011. In addition, a supplement of $21.8 billion was also injected to establish new universities, research labs and scholarship programs for higher education. In Dec 2012, the educational budget reached the highest-ever level of $54.54 billion for year 2013 [2]. The GCC countries with the highest per capita GDP are Qatar ($75175.82); Kuwait ($50566.56), United Arab Emirates ($44544.27); Saudi Arabia ($24246.52); Bahrain ($18867.55), while their spending on R&D is as follows: Qatar 2.7%; Saudi Arabia 0.3%; United Arab Emirates 0.15%; Bahrain 0.2% and Kuwait 0.09% [2].

Most of the GCC universities are collaborating with science and talent rich institutes to establish international collaboration to promote research culture. Sadly, the research out come in medical education from these countries is greatly lacking.

Hamdy et al. [10] reported that, in Gulf Cooperation Council (GCC) countries there is a swift and major social, cultural, and economic transformation, however, the development of medical education in the region is comparatively new. They reported that, few medical schools are reviewing their curriculum while newly established medical schools are developing their programs following current trends in medical education, particularly problem-based learning and integrated curricula. The authors also reported that there is lack of faculty development in medical education, shortages of faculty and availability of clinical training facilities. We believe that, these are the factors that contribute to the lack of research in medical education.

Khalid bin Abdulrehman [11] demonstrated that, in GCC, since the last two decades, the curriculum in medical education has changed to maintain its efficiency and effectiveness. The author stated that, majority of medical schools 43.3% are located in Saudi Arabia. 40% of the colleges followed the traditional curriculum, while the remaining 60% followed a hybrid problem-based learning (PBL) curricula. The majority of the traditional colleges and the recently established medical schools are adopting the hybrid PBL curricula. Even with the change in curriculum the GCC countries did not produce sufficient research in medical education.

In agreement to our findings, Khalid bin Abdulrehman [12] reported that in Saudi Arabia medical professionals have made obvious contributions in scientific research but research in medical education is still lacking. The author conducted a PubMed database research for papers relating to medical topics in Saudi Arabia, and found only 461 (0.02%) papers in medical education from Saudi Arabia. The author reported the numerous factors which hamper the medical education research in Saudi Arabia. The factors limiting research in medical education includes (i) lack of funding in medical education (ii) biomedical industries have little commercial interest in medical education (iii) lack of research skills (iv) limited numbers of peer-reviewed journals in medical education (v) lack of an academic recognition in promotions and hiring. Moreover, medical educationists are focusing more in biomedical and clinical research rather than in medical education. Our findings are in agreement to the results of Khalid bin Abdulrehman [12] that Saudi Arabia contributed enough number of research publications in bio-medical sciences in ISI-web of science journals, however, in medical education Saudi Arabia produced very little 323 (0.79%) research papers in ISI web of science and in medical education the Hirsch index (*h*-index) of Saudi Arabia is 14. In the present study, we took one step forward to the study of Khalid bin Abdulrehman [12], they gathered the research publications based on pub med database, however, in the this study, the source of information is ISI-web of science, a more reliable and valid source of science bibliometric indicators.

Choung and Hwang [13] reported that universities play an important role in increasing number of research papers in the ISI database and the related research activities. Total number of universities in GCC countries is 126 and out of them 40 are medical universities/colleges. Since, the number of universities were increased, the number of research paper were also significantly increased. This is an established fact that the basic birth place of the research publications is the universities. Meo et al. [14] reported that, the countries with large number of universities were producing more research papers. Similarly, in the present study, we found that, GCC-countries have increased the number of universities and degree awarding institutes and consequently, research outcome in medical education has also increased.

In another study, Meo et al. [3] reported that, the Middle East countries who spend more on Research and Development (R&D) produced better research outcomes in pharmacological sciences. Similarly, in the present study, we found that, the GCC countries which are spending more on education and research, produced larger number of research papers in science but not in medical education. GCC countries contribution to medical education research papers remains very small.

### Study strengths and limitations

The strengths of this study are: we recorded the information regarding GCC countries performance in research from very reliable source, Institute of Scientific Information (ISI), Web of Science, Journal Citation Reports (Thomson Reuters). However, the limitation of the present study is that occasionally citation count tools may mis-cite or re-cite a paper, and there are chances of the same paper appearing twice with slightly different details. This may affect the number of documents or citation counts.

## Conclusions

GCC countries contributed very little research in medical education during the period 1996–2013. They introduced many incentives but their progressive impact in medical education research is still marginalized.

### Recommendations

GCC countries must improve their research outcomes in medical education to produce better physicians and to enhance the standards in medical practice. GCC states need to invest more in R&D in the field of medical education, establish collaboration with talent and research rich international institutes. The authorities should provide more research grants, rewards for researchers, enrollment of postgraduate and PhD students. Medical schools should establish research and evidence based curricula and engage both undergraduate and postgraduate medical students in medical education scholarly research activities. They should offer elective and compulsory research projects in medical education for their graduation. Students should be provided with an opportunity to present their research results in paper or poster presentation in science conferences in general and medical education conferences in particular. It will provide foundations for strong approach to learning and understanding of the challengeable issues in medical education to solve the problems along with socializing the students in medical science community where the scholarship of discovery is acknowledged and valued.

## References

[1] Cooperation Council for the Arab States of the GulfAvailable at: http://en.wikipedia.org/wiki/Cooperation_Council_for_the_Arab_States_of_the_Gulf, cited date Bon 12, 2013.

[2] Saudi Arabia’s golden age of learning: Available at: http://www.arabnews.com/saudiarabia%E2%80%99s-%E2%80%98golden-age-learning%E2%80%99-under-king-Abdullah, retrieved on Feb 16, 2013.

[3] Meo SA, Usmani AM, Vohra MS, Bukhari IA (2013). Impact of GDP, Spending on R&D, number of universities and scientific journals on research publications in pharmacological sciences in Middle East. Eur Rev Med Pharmacol Sci.

[4] Durieux V, Gevenois PA (2010). Bibliometric indicators: quality measurements of scientific publication. Radiology.

[5] Preis T, Moat HS, Stanley HE, Bishop SR (2012). Quantifying the advantage of looking forward. Sci Rep.

[6] Macilwain C (2010). What science is really worth. Nature News.

[7] International association of universities, the list of universities of the world. Available at: in http://www.iau-aiu.net/content/list-heis, Retrieved on Nov 26 2013.

[8] Journal citation report, ISI web of Knowledge. Available at: http://webofknowledge.com/JCR/JCR?PointOfEntry=Home&SID=4FeKpokbnHkLlmE1OGe, Retrieved on Oct, 2, 2013.

[9] SCI-mago Journal and country ranking. Available at: http://www.scimagojr.com/ retrieved on Oct 2, 2013.

[10] Hamdy H, Telmesani AW, Al Wardy N, Abdel-Khalek N, Carruthers G, Hassan F (2010). Undergraduate medical education in the Gulf Cooperation Council: a multi-countries study (Part 1). Med Teach.

[11] Khalid BA (2008). The current status of medical education in the Gulf Cooperation Council countries. Ann Saudi Med.

[12] Bin Abdulrahman KA (2012). The value of medical education research in Saudi Arabia. Med Teach.

[13] Choung JY, Hwang HR (2000). National systems of innovation: Institutional linkages and performances in the case of Korea and Taiwan. Scientometrics.

[14] Meo SA, Abeer Al Masri A, Usmani AM, Memon AN, Zaidi SZ (2013). Impact of GDP, spending on R&D, number of universities and scientific journals on research publications among Asian countries. PLoS One.

